# Supplementary material for: From sequence to enzyme mechanism using multi-label machine learning
Source: BMC Bioinformatics. 2014 May 19;15:150. doi: 10.1186/1471-2105-15-150 (PMC4229970; doi:10.1186/1471-2105-15-150)
Supplement: Additional file 2 — Java code of ml2db. Additional file ml2db_code.tar.gz contains the Java source code to run the multi-label machine learning experiments and save the results to database. The code’s Javadoc is included. [file 1471-2105-15-150-S2.zip › additional file 2/ml2db/ml2db/doc/mulan/data/DataUtils.html]

DataUtils


JavaScript is disabled on your browser.


- Overview
- Package
- Class
- Use
- Tree
- Deprecated
- Index
- Help

- Prev Class
- Next Class

- Frames
- No Frames

- All Classes

- Summary:
- Nested |
- Field |
- Constr |
- Method

- Detail:
- Field |
- Constr |
- Method


mulan.data

## Class DataUtils

- java.lang.Object
- - mulan.data.DataUtils

- ---

    

  ```
  public class DataUtils
  extends java.lang.Object
  ```

  Utility class for data related manipulation functions.

  Author:
  :   Jozef Vilcek

- - ### Constructor Summary

    Constructors

    Constructor and Description

    `DataUtils()`
  - ### Method Summary

    Methods

    | Modifier and Type | Method and Description |
    | --- | --- |
    | `static weka.core.Instance` | `createInstance(weka.core.Instance typeProvider, double weight, double[] attrValues)` Creates a new `Instance`. |
    | `static weka.core.Instance` | `createInstance(weka.core.Instance typeProvider, int numAttributes)` Creates a new `Instance`. |

    - ### Methods inherited from class java.lang.Object

      `equals, getClass, hashCode, notify, notifyAll, toString, wait, wait, wait`

- - ### Constructor Detail


    - #### DataUtils

      ```
      public DataUtils()
      ```
  - ### Method Detail


    - #### createInstance

      ```
      public static weka.core.Instance createInstance(weka.core.Instance typeProvider,
                                      double weight,
                                      double[] attrValues)
      ```

      Creates a new `Instance`. The actual type is determined based on passed instance object.

      Parameters:
      :   `typeProvider` - the instance from which type for new instance is determined
      :   `weight` - the weight of a new instance
      :   `attrValues` - attribute values for a new instance

      Returns:
      :   A new `Instance`.


    - #### createInstance

      ```
      public static weka.core.Instance createInstance(weka.core.Instance typeProvider,
                                      int numAttributes)
      ```

      Creates a new `Instance`. The actual type is determined based on passed instance object.

      Parameters:
      :   `typeProvider` - the instance from which type for new instance is determined
      :   `numAttributes` - number of attributes for new instance

      Returns:
      :   A new `Instance`.


- Overview
- Package
- Class
- Use
- Tree
- Deprecated
- Index
- Help

- Prev Class
- Next Class

- Frames
- No Frames

- All Classes

- Summary:
- Nested |
- Field |
- Constr |
- Method

- Detail:
- Field |
- Constr |
- Method
